# 

**Published:** 1848-04

**Authors:** 


### OHIO COLLEGE OF DENTAL SURGERY.

The lectures commence annually in this institution, 1st Monday of November, and close last of February—cost of full course about $100.

Arrangements are in progress for a reorganization of the school, rendered necessary by the resignation of Prof. Rogers. It is expected some changes will be made very much for the benefit of the school, and which will afford to the student facilities for the acquisition of practical and theoretical knowledge, unsurpassed any where.

Of the fact that such an institution is needed at the West, we need no greater proof than that afforded at the last session, (notwithstanding the resignation of Prof. Cook, only two days before the lectures commenced,) yet some eight or nine students stepped forward for a regular course, and by the 20th, the time for closing our matriculation book, some five or six more had made known their intention to spend the winter with us. Owing, however, to our prolonged illness at that time, our class was rendered much smaller than it would otherwise have been. At present some twelve to fifteen applications stand ready for next session. We assure these and all others, that we shall be far better prepared next winter to receive and do justice to a large class than we have ever been. Two or three years is necessary to complete an organization of this kind, and make such arrangements as it regards mode of instruction, in the Labratory, in the Infirmary, and arrangement of Lectures, as may be most advantageous to the student.

We hope, before the issuing of the next number of the Register, to present such an organization as shall secure to this school a liberal support from the Profession, and place it on such a permanent footing as shall command the approval of the intelligent of all professions.

### SUBSCRIBERS,

May rest assured that on the publication of each number, it will be mailed to them. If they come not to hand, a note, postage paid, informing us of the fact, will receive attention by the forwarding of the number.—CIN. ED.

---

### DR. IDE.

This gentleman, favorably known to us for several years past, and whose residence for the past year in our city, served to increase our esteem, we are sorry to say, has returned to his former field of labor. We are certain that his friends of Zanesville gave him up at first with great reluctance, not greater however, we feel, than did ourself and the friends he had made in this city. Go where you will, friend Ide, may health and prosperity attend you.

Owing to the Dr.'s removal, his communication on full setts of teeth will be delayed till our next.

---

### DR. E. TAYLOR'S ESSAYS ON FILLING TEETH.

We are sorry that they could not be continued in this number, but circumstances have been such as to prevent the preparatton of an article on this subject. We have the promise of its continuance for our next.—C. ED.

